# PTPN23 Overexpression Is Associated with Reduced Proliferation and Tumor Growth in Murine Models of Colorectal Cancer

**DOI:** 10.3390/ijms27177955

**Published:** 2026-09-07

**Authors:** Rocio Sanchez Alvarez, Claudia Gottier, Ana Montalban-Arques, Anna Bircher, Doris Pöhlmann, Marlene Schwarzfischer, Anne Müller, Silvia Lang, Marianne R. Spalinger, Michael Scharl

**Affiliations:** 1 Department of Gastroenterology and Hepatology, University Hospital Zurich, University of Zurich, 8091 Zurich, Switzerland; rocio.sanchez@usz.ch (R.S.A.);; 2 Institute of Molecular Cancer Research, University of Zurich, 8057 Zurich, Switzerland

**Keywords:** PTPN23 overexpression, EGFR, reduced tumor size, tumor suppression

## Abstract

Protein tyrosine phosphatases (PTPs) are key regulators of intracellular signaling cascades involved in cell growth, proliferation, differentiation, inflammation, and cancer. PTPN23, a non-receptor PTP, is involved in endosomal sorting and thereby regulates the internalization of growth factor receptors, such as the epidermal growth factor receptor (EGFR). PTPN23 has been proposed to exert tumor-suppressive effects in several epithelial cancers, including breast and lung cancer. PTPN23 is ubiquitously expressed in the body; however, it is particularly highly expressed in the intestine, where it plays an essential role in maintaining intestinal homeostasis. Despite these observations, its involvement in colorectal carcinoma (CRC) remains largely unexplored. To investigate the function of PTPN23 in CRC, we generated PTPN23-overexpressing CRC cell lines and evaluated their effects in vitro and in subcutaneous tumor models. Our in-vitro findings demonstrate that PTPN23 overexpression resulted in reduced proliferation levels and follow-up experiments showed suppression of colorectal cancer growth and proliferation in experimental CRC models. These effects were accompanied by changes in EGFR-associated signaling; however, the underlying mechanism, including a potential role of EGFR trafficking, remains to be established through direct experimental validation. Together, these findings expand current knowledge of PTPN23 in colorectal cancer and provide a basis for future studies addressing its biological function and therapeutic relevance.

## 1. Introduction

Protein tyrosine phosphatases (PTPs) regulate signaling pathways by dephosphorylating tyrosine residues on proteins [1]. The *PTP-Non receptor type 23* (*PTPN23*) gene encodes a pseudophosphatase that functions independently of phosphatase activity due to a mutation in its catalytic site [2]. Moreover, PTPN23 has been identified as a component of the endosomal sorting complexes required for transport (ESCRT) [3]. The ESCRT mediates the internalization of specific cell surface receptors involved in processes such as proliferation, differentiation, adhesion, motility, invasion, migration, apoptosis and endocytosis, including the epidermal growth factor receptor (EGFR) [1,3,4]. Disruption in endosomal sorting has been implicated in the stabilization of cell surface receptors and persistent signal transduction, which are well-known hallmarks of cancer [1,5,6]. For instance, excessive EGFR signaling can drive tumorigenesis, as demonstrated in lung, cervical and breast cancer, as well as in glioblastoma [6,7].

Epithelial cancers including kidney, breast, lung, and cervical tumors frequently exhibit hemizygous mutations or deletions in the chromosomal region where PTPN23 is located. Moreover, decreased PTPN23 mRNA expression has been associated with poor prognosis and reduced survival rates in lung and breast cancers [3].

Although *PTPN23* is expressed ubiquitously in adult tissues, its expression is particularly high in the gastrointestinal tract, reflecting its intestinal regulatory role [8]. Despite its implication in epithelial cancers and its essential role in intestinal homeostasis, the potential involvement of PTPN23 in CRC development remained largely unexplored. This is particularly important because CRC remains one of the most common cancers worldwide and continues to be associated with high mortality rates [9]. Since treatment options are limited, new therapeutic approaches are urgently needed [10,11].

We demonstrate that PTPN23 overexpression reduces CRC cell proliferation and tumor growth in experimental models, supporting a growth-inhibitory role for PTPN23 in colorectal cancer cells.

## 2. Results

### 2.1. PTPN23 Is Associated with CRC Cell Dependency and EGFR-Related Signaling in Human Datasets

**Figure 1:**
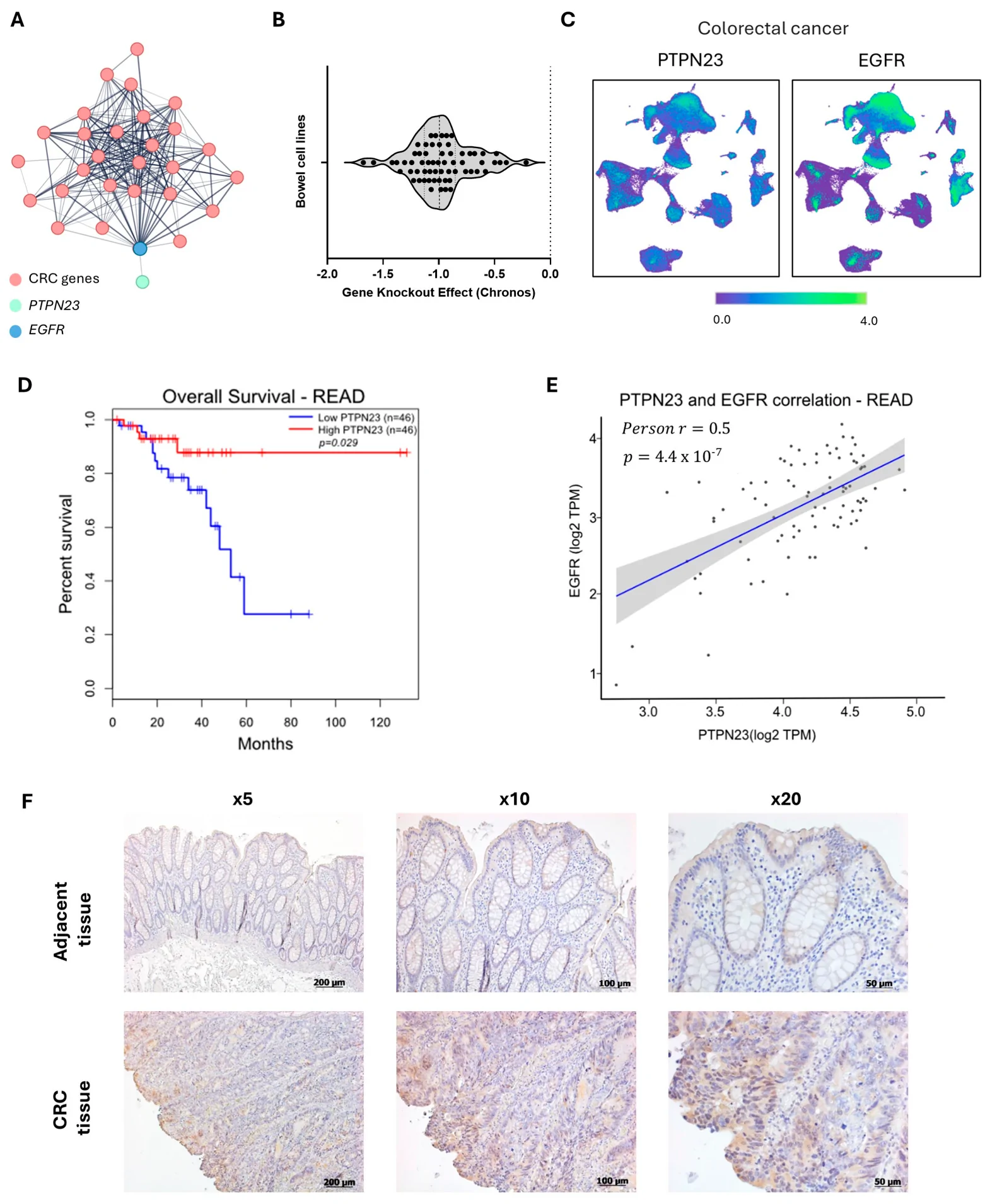
PTPN23 expression in human CRC reveals EGFR-associated dependencies. (**A**) STRING protein–protein interaction network of CRC-related genes (red) and PTPN23 (green). EGFR indicated in blue. (**B**) DepMap of PTPN23 gene effect in human CRC cell lines. (**C**) UMAP plot showing the expression of PTPN23 and EGFR in a human CRC single-cell RNA sequencing atlas. (**D**) Survival curve from rectal adenocarcinoma patients stratified into high (red) or low (blue) PTPN23 expression levels (TCGA database). (**E**) Correlation between PTPN23 and EGFR expression levels in rectal adenocarcinoma patients (TCGA database). Each dot represents a single patient, plotting its EGFR against PTPN23 expression values. The trend line illustrates the overall positive linear relationship between the two genes and gray area represents the confidence interval. (**F**) PTPN23 staining in colon tumor tissue and surrounding healthy tissue within the same patient (*n* = 3, biological replicates) (50×, 100×, 200× magnification).

PTPs have attracted significant attention in the context of cancer due to their role in cell death and survival [4,12]. Among other PTPs, the deletion of PTPN23 has been detected in some epithelial cancers [3]. Despite its well-described tumor suppressive role in breast or lung cancer, and its high expression in intestinal epithelial cells, the role of PTPN23 in CRC is poorly characterized [3,8]. In this regard, computational predictions from the STRING database indicated a connection between PTPN23 and CRC-associated genes (Figure 1A). This predicted network included EGFR and signaling pathways related to p53, ErbB, MAPK, and apoptosis. Consistent with these observations, genome-wide CRISPR screens (DepMap) revealed that *PTPN23* is required for the viability of human cancer cells, including CRC cell lines (Figure 1B, Supplementary Figure S1A). Similar observations were obtained from the analysis of a public single-cell CRC reference atlas [13]. Although PTPN23 was broadly expressed, it showed marked overlap with the EGFR-expressing cell cluster (Figure 1C), an observation that is consistent with previously reported associations between PTPN23 and EGFR signaling [1]. Interestingly, this specific cell cluster consisted of malignant cells from patients with CRC (Supplementary Figure S1B), suggesting a possible tumor-specific role for PTPN23.

We next studied the PTPN23 expression and the survival data from TCGA dataset. In this dataset, CRC is separated into rectal adenocarcinoma (READ) and colon adenocarcinoma (COAD) patients. COAD patients with high expression of PTPN23 showed a slight improvement in the survival percentage in the first 50 months but this effect was not sustained in the following months (Supplementary Figure S1C). On the other hand, READ patients with high PTPN23 expression exhibited significantly improved overall survival compared with those in the low-expression group (Figure 1D). In these patients, PTPN23 expression was positively correlated with EGFR expression (Figure 1E).

To confirm the in-silico findings, we performed PTPN23 staining on primary colon tumor tissues and compared them with non-tumorous tissues from the same patients as an exploratory dataset. CRC tissues showed higher PTPN23 expression than the surrounding non-tumorous tissues from the same patients (Figure 1F). PTPN23 staining was predominantly localized to intestinal epithelial cells, consistent with its previously reported expression pattern. These findings support an association between PTPN23 expression and colorectal cancer although its functional role in human tumors would require validation.

### 2.2. Decreased Metabolomic Activity and Growth of CRC Cells Were Detected upon PTPN23 Overexpression

**Figure 2:**
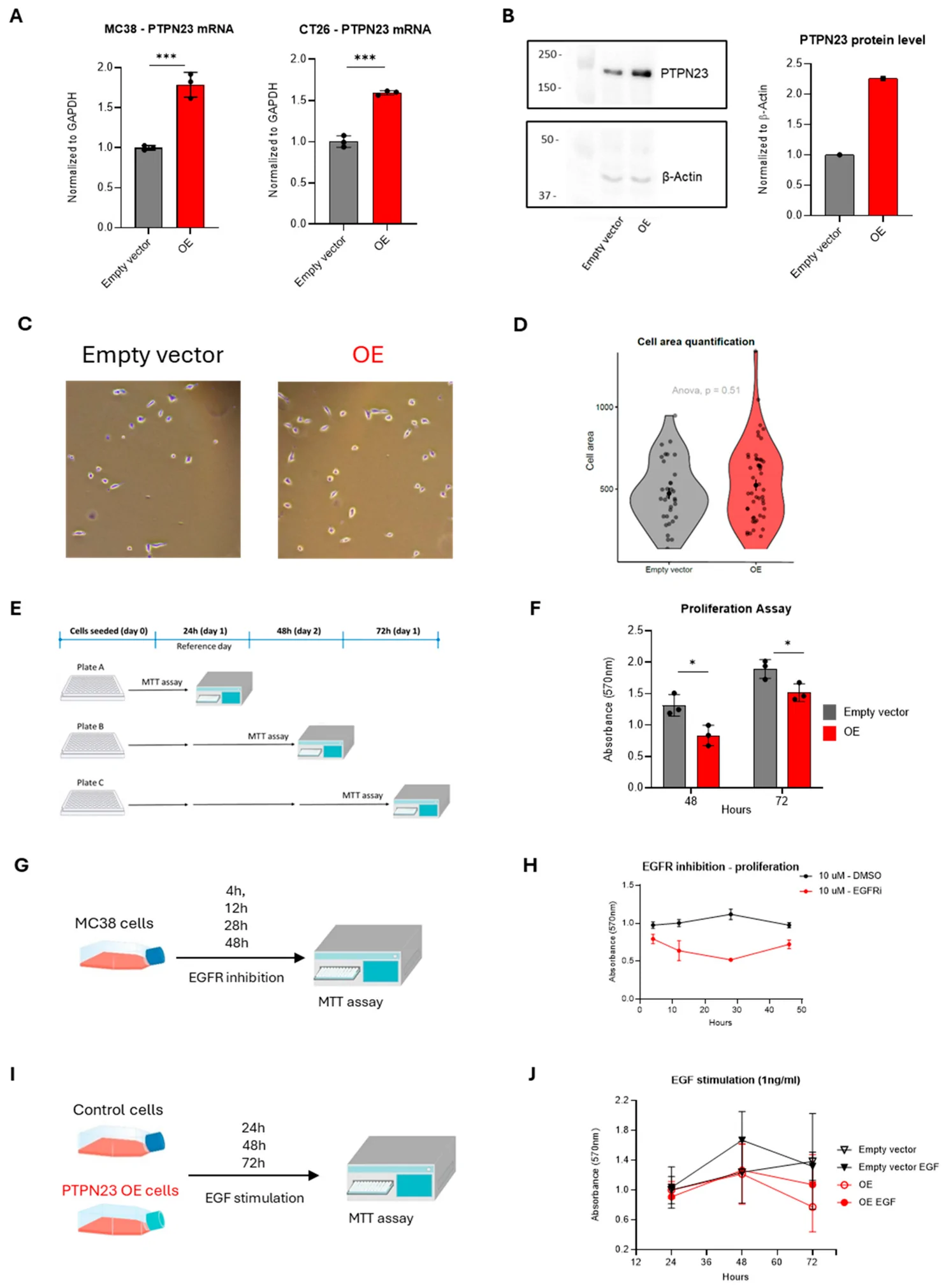
PTPN23 overexpression is associated with reduced cellular metabolic activity and proliferative activity. (**A**) PTPN23 mRNA expression level in transduced MC38 and CT26 cells using an empty vector (control) or PTPN23-overexpressing vector. Three independent experiments were performed. One representative experiment shown. (**B**) Western blot and protein quantification of PTPN23 (179 kDa) from transduced MC38 cells. β-Actin (42 kDa) was used as a loading control. One experiment performed. (**C**) Representative images of transduced MC38 cells. (**D**) Quantification of cell area in control and OE groups. Quantification performed using ImageJ. (**E**) Experimental design of the MTT assay using transduced MC38 cells. (**F**) Proliferation rates of OE MC38 cells (*n* = 4) or empty vector cells (*n* = 4) at 48 h or 72 h post-seeding, normalized to 24 h values. Three independent experiments performed. One representative experiment shown. (**G**) Experimental design for EGFR inhibition in non-transduced MC38 cells. (**H**) Proliferation level of MC38 cells treated with 10 µM of EGFR inhibitor (*n* = 4) or vehicle treatment (*n* = 4). (**I**) Experimental design of EGF stimulation as a rescue experiment using transfected MC38 cells. (**J**) Proliferation rate of transduced MC38 cells stimulated with EGF (*n* = 4) or with vehicle control (*n* = 4). Three independent experiments were performed. Pooled data shown. Error bars represent mean ± SD. *p*-values were determined by 2-tailed Mann–Whitney U Test (* *p* < 0.05, *** *p* < 0.001).

To functionally examine the role of PTPN23 in the context of CRC, we engineered the murine colon cancer cell lines MC38 and CT26 to overexpress PTPN23. Previously, the baseline expression levels of *PTPN23* across different cancer types (Supplementary Figure S2A) were examined, revealing that CT26 and MC38 cell lines exhibited the lowest and highest expression levels, respectively. Both lines were thus transduced with a PTPN23-overexpressing construct or an empty vector as control and selected by using antibiotic-containing selection media. By qPCR, we confirmed that the levels of *PTPN23* mRNA were significantly higher in the overexpressing conditions in MC38 and CT26 cells (Figure 2A). Consistent with the changes in mRNA levels, the overexpressing condition showed a two-fold increase in the PTPN23 protein level compared to the control group in MC38 cells (Figure 2B). These results confirmed efficient overexpression of PTPN23 in the transgenic cells.

Once PTPN23-overexpression was confirmed, we next studied the features of the transduced cells in vitro. As PTPN23 had previously been reported to affect many different cell pathways as well as cell adhesion proteins [14], we assessed possible morphological differences between the two groups. However, no detectable differences in cell morphology were observed between the two groups (Figure 2C,D, Supplementary Figure S2B). Therefore, PTPN23 overexpression did not impact cell morphology.

During culture, PTPN23-overexpressing cells required longer intervals between passages, and stable overexpression was progressively lost over time. These observations are consistent with a proliferative disadvantage associated with PTPN23 overexpression. Therefore, we conducted an MTT assay as an indirect measure of cellular metabolic activity (Figure 2E). Metabolic activity was significantly lower in the PTPN23-overexpressing group than in the control group (Figure 2F), consistent with reduced proliferative activity. Together, these findings indicate that PTPN23 overexpression is associated with reduced cellular metabolic activity and reduced proliferative activity.

We next assessed whether EGFR signaling could contribute to the phenotype observed after PTPN23 overexpression, rather than seeking to establish EGFR dependence. To this aim, we first investigated whether EGFR blockade in non-transduced MC38 cells mimics the proliferation defect observed in overexpressing cells (Figure 2G). Our results showed that cells treated with Erlotinib, a commonly used EGFR inhibitor [15,16], displayed significantly lower values after 24h of treatment compared with the vehicle-treated cells (Figure 2H, Supplementary Figure S2C). This observation was consistent with our previous results and the literature [1,8], supporting a role for EGFR in regulating CRC cell proliferation.

To further explore a possible association between PTPN23 overexpression and EGFR signaling, we conducted a rescue experiment by stimulating the transduced cells with EGF (Figure 2I). Prior to treatment, cells were serum-starved to synchronize the cell population and avoid background. After 72 h of EGF stimulation, overexpressing cells exhibited a partial recovery of proliferation compared to untreated overexpressing cells, which showed the lowest cell growth levels (Figure 2J). Notably, the high variance within groups resulted in a lack of statistical significance.

### 2.3. PTPN23-Overexpressing Tumors Show Significantly Reduced Tumor Sizes

**Figure 3:**
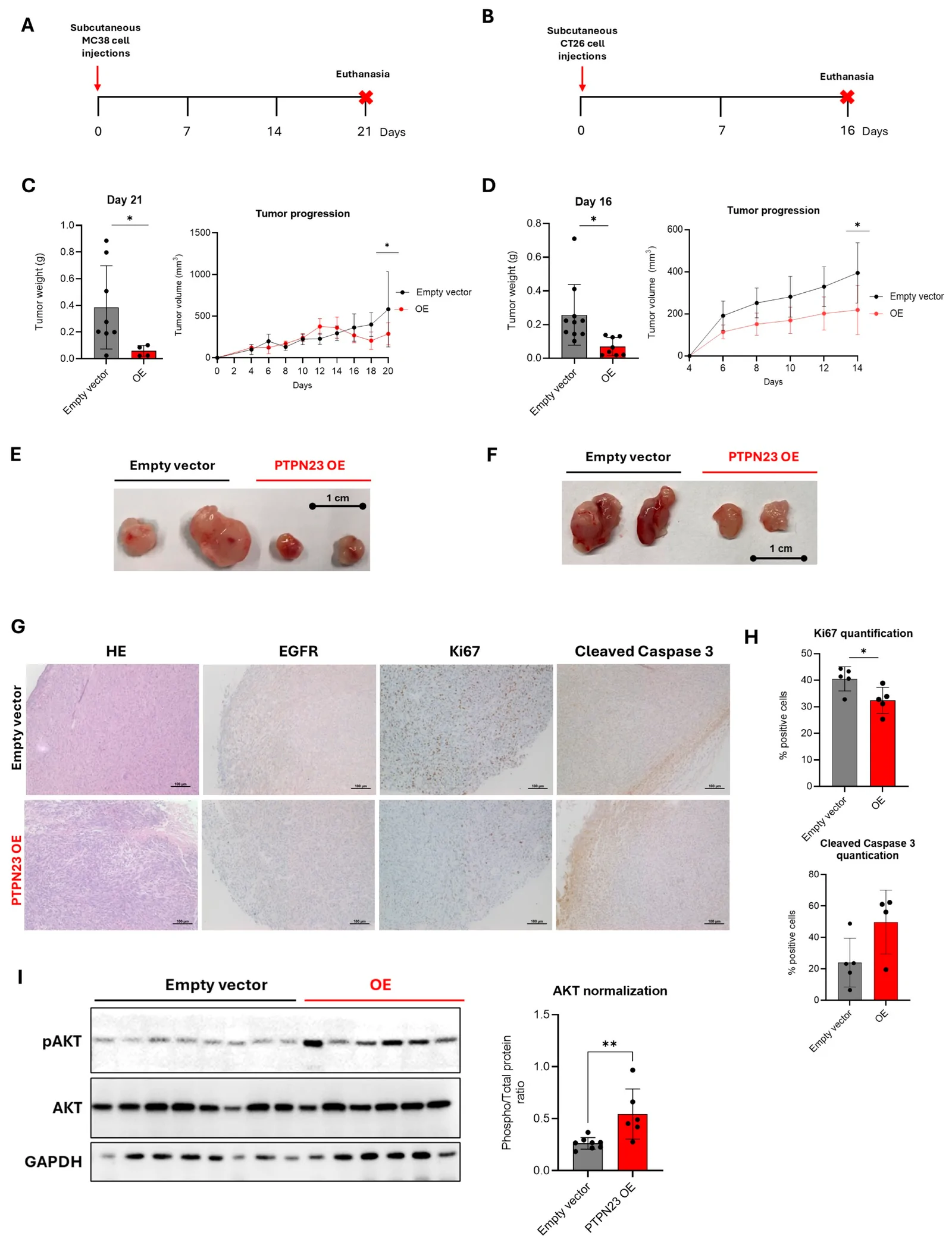
PTPN23-overexpressing tumors result in smaller tumor size and slower tumor growth. (**A**) Experimental design of subcutaneous injection of transduced MC38 cells into C57BL/6 mice (250,000 cells/injection diluted 1:2 in Matrigel). Data from two independent experiments; control group (*n* = 5) and PTPN23-overexpressing group (*n* = 5). One representative experiment shown. (**B**) Experimental design of subcutaneous injection of transduced CT26 cells into BALB/c mice (500,000 cells/injection diluted 1:2 in Matrigel). Data from two independent experiments; control group (*n* = 5) and PTPN23-overexpressing group (*n* = 5). One representative experiment shown. (**C**) MC38 tumor weight at termination and tumor volume over time. (**D**) CT26 tumor weight at termination and tumor volume over time. (**E**) Representative images of isolated MC38 tumors on day 21. (**F**) Representative images of isolated CT26 tumors on day 14. (**G**) H&E and IHC staining for cleaved Caspase-3, Ki67, and EGFR in CT26 tumors (100× magnification). The staining was performed using tumors from one independent experiment (control, *n* = 8; PTPN23-overexpressing, *n* = 6). (**H**) Quantification of cleaved Caspase-3 and Ki67 staining. (**I**) Western blot and protein quantification of total protein AKT (60 kDa) and phosphorylated-AKT (60 kDa) from CT26-tumor model from empty-vector group (*n* = 8) and PTPN23-overexpressing group (OE; *n* = 6). GAPDH (37 kDa) was used as a loading control. One experiment performed. Error bars represent mean ± SD. *p*-values were determined by 2-tailed Mann–Whitney U Test (* *p* < 0.05, ** *p* < 0.01). For tumor growth curves, statistical analysis was performed on the final tumor volume measurement.

Given the effects on cell proliferation in our in vitro studies, subcutaneous tumor cell injection experiments were performed to evaluate the impact of PTPN23 overexpression in vivo. We therefore injected transduced MC38 or CT26 cells subcutaneously into the flanks of C57BL/6 mice or BALB WT mice, respectively (Figure 3A,B). In both injection experiments, tumor growth in the overexpressing group was clearly lower compared to the control group (Figure 3C,D), as represented by significantly smaller tumors in the mice receiving PTPN23 overexpressing cell injections (Figure 3E,F, Supplementary Figure S3A,B). Notably, two animals from the MC38 PTPN23-overexpression group died during the experiment at day 14. No evidence that the deaths were related to the experimental procedures was observed and no other adverse events were reported.

To investigate the molecular changes underlying the reduced tumor size observed in the overexpressing group, we performed both general and specific stainings for EGFR, cleaved Caspase-3, and Ki67 (Figure 3G, Supplementary Figure S3C). No differences were seen in the H&E staining between the overexpressing and the control tumors (Figure 3G). EGFR staining revealed a slight reduction in expression in the overexpressing tumors compared to the control group (Figure 3G). The overexpressing tumors displayed a lower number of Ki67+ cells indicating a reduced proliferation, which corroborates our in vitro findings (Figure 3G,H). When assessing apoptosis, the overexpressing tumors showed a trend towards increased cleaved Caspase-3 positive cells compared with the control group, suggesting enhanced apoptosis in overexpressing tumors (Figure 3G,H). We next assessed the phosphorylation of the EGFR downstream effectors AKT and ERK. No significant difference was observed in the p-ERK/ERK ratio (Supplementary Figure S3D). In contrast, AKT phosphorylation was significantly increased in the overexpression group (Figure 3I). These findings indicate altered activation of the AKT pathway following PTPN23 overexpression. Whether this reflects changes in EGFR trafficking or other signaling mechanisms cannot be determined from the present data.

### 2.4. Overexpression of PTPN23 Is Not Associated with Changes in Tumor Immune Cell Infiltration

**Figure 4:**
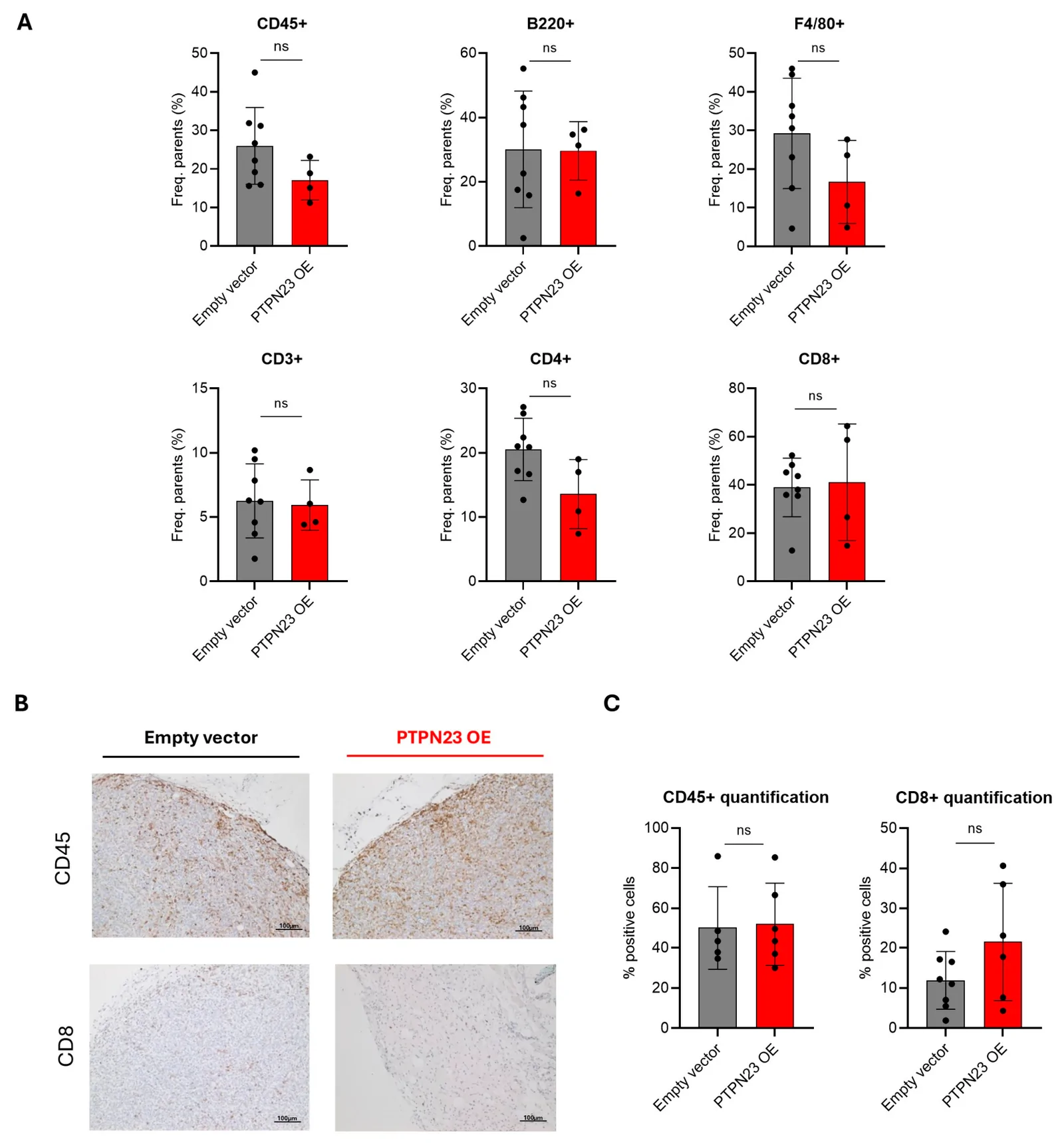
PTPN23-overexpressing CT26 cell injection does not affect the immune response. (**A**) Frequencies of parents of CD45+, B220+, F4/80+, CD3+, CD4+ T cells and CD8+ T cells from tumor tissue. (**B**) Pictures of CD45+ and CD8+ IHC stainings of CT26 tumors from mice with control or OE cells. (**C**) IHC staining for CD45+ and CD8+ in CT26 tumors from control or PTPN23-overexpressing groups (100× magnification). Data from one experiment consisting of control group (*n* = 5) and PTPN23 overexpressing group (*n* = 5). Error bars represent mean ± SD. *p*-values were determined by 2-tailed Mann–Whitney U Test (ns = non-significant).

Given the pronounced effect of PTPN23 overexpression on tumor growth in the in vivo experiments, we investigated whether the tumor-infiltrating immune cells were involved in the recognition of transduced cells. For this purpose, we performed flow cytometry analysis of CD45+ immune cells isolated from the tumor tissue of both PTPN23 WT and overexpressing tumors. The immune markers used in the analysis did not reveal any relevant difference between the groups (Figure 4A, Supplementary Figure S4A). To confirm these results, we conducted IHC staining for immune markers on the tumor samples. No differences were detected in the total number of leukocytes (CD45+) or cytotoxic T-cell (CD8+) infiltration (Figure 4B,C, Supplementary Figure S4B). These data do not provide evidence for major differences in the analyzed immune-cell populations between control and PTPN23-overexpressing tumors. However, the limited flow-cytometry dataset and immune stainings preclude excluding more subtle immune-mediated effects.

In conclusion, our results indicate that overexpression of PTPN23 in CRC results in reduced tumor size and proliferation in experimental CRC models, supporting further investigation of PTPN23 in colorectal cancer biology.

## 3. Discussion

By investigating the effects of PTPN23 in experimental CRC models, we found that PTPN23 overexpression resulted in reduced proliferation rates, which collectively led to a significant decrease in tumor size.

The analyzed human CRC datasets and CRC tissue staining indicated elevated expression of PTPN23 in malignant cells compared with non-malignant cells. Additionally, the PTPN23 expression levels found in CRC patients in the datasets showed substantial variability. The TCGA overall survival rates, particularly in READ, showed that higher PTPN23 expression was associated with improved survival, whereas lower PTPN23 expression was associated with poorer outcomes. One possibility is that increased PTPN23 expression represents a compensatory response to elevated EGFR signaling. This is in line with the STRING result, which predicted a link between PTPN23 and CRC markers through EGFR. However, this hypothesis remains unexplored and requires direct experimental validation.

Given the association between PTPN23 expression and patient outcomes, we next considered the potential mechanisms through which PTPN23 may exert a growth-inhibitory role in CRC. Dysregulation of EGFR is a well-established driver of CRC progression [17] and, while EGFR mutations are common in other cancer types, CRC typically exhibits a 3–5-fold amplification of EGFR or impaired endocytosis resulting in sustained pathway activation [18,19]. Recent studies support this mechanism: the deletion of PTPN23 in cervical carcinoma cells disrupts ESCRT-mediated EGFR trafficking, stabilizing the receptor at the cell surface and enhancing downstream signaling [6]. Similarly, another study in human colonic cancer cells demonstrated that PTPN23 expression significantly reduced EGFR signaling upon EGF stimulation [1]. Moreover, downstream EGFR signaling is closely associated with proliferative markers such as Ki67 [20,21]. Overexpression of both EGFR and Ki67 has been associated with high tumor burden and poor prognosis in several cancers [20,21]. Additionally, the increased phosphorylation of AKT observed in our study may reflect a context-dependent role of AKT signaling. Beyond its classical role in promoting cell survival, AKT has also been reported to participate in EGFR trafficking and degradation through endosomal pathways involving lysosomal targeting and ESCRT-dependent sorting machinery [22]. In contrast, we did not observe statistically significant changes in ERK phosphorylation. This may reflect the ability of ERK signaling to be maintained through alternative upstream signaling pathways or compensatory mechanisms independent of EGFR modulation [23,24]. Together, these findings suggest distinct regulatory effects of PTPN23 on AKT and ERK pathways and highlight the potential involvement of additional signaling mechanisms underlying the observed phenotype. Although our observations are consistent with previous reports describing a role for PTPN23 in EGFR trafficking, our study does not directly demonstrate altered EGFR internalization. The partial, non-significant rescue following EGF stimulation and the observed changes in AKT phosphorylation should therefore be interpreted as indirect evidence of altered EGFR-associated signaling rather than proof of a specific trafficking mechanism. Direct assessment of EGFR internalization and receptor dynamics will be required to determine whether this pathway underlies the reduced proliferation observed in our models.

On the other hand, our findings reveal an apparent contradiction. Whereas PTPN23 overexpression suppresses tumor growth in our experimental models, large-scale dependency datasets indicate that PTPN23 is required for cancer cell viability. Although recent work has proposed that PTPN23 may simultaneously dampen oncogenic signaling while preventing receptor accumulation-associated cell death [4], our data do not directly address this possibility. Consequently, the biological basis for the apparent discrepancy between tumor suppressive effects and cellular dependency remains unresolved and represents an interesting question for future investigation. Moreover, identifying new targets that modulate cell death could significantly advance cancer research and treatment, particularly in CRC, where current therapies are often ineffective [25]. These findings suggest that the role of PTPN23 in regulating cell survival and death pathways warrants further investigation, particularly in the context of CRC therapy resistance.

In conclusion, our study demonstrates that PTPN23 overexpression suppresses proliferation and tumor growth in CRC models and is associated with alterations in proliferation-related signaling. These findings support further investigation of the role of PTPN23 in colorectal cancer biology, including its relationship with ESCRT-mediated trafficking.

### Limitations

Our study has several limitations that should be considered. First, the mechanistic association between PTPN23 and EGFR signaling was assessed through indirect evidence and supported by previous literature; direct experiments demonstrating enhanced EGFR internalization were not performed in the present study. Consequently, the proposed link between PTPN23 and altered EGFR trafficking remains hypothetical and requires direct experimental validation.

Second, our attempts to generate PTPN23 knockout cells using CRISPR-Cas resulted in complete loss of viability, limiting our functional studies to overexpression models. Although our findings are consistent with growth-suppressive effects of PTPN23 in experimental CRC models, public dependency datasets indicate that PTPN23 is also required for cancer cell viability. The biological basis for this apparent paradox remains unresolved and was not directly addressed in the present study. Additionally, the use of an orthotopic model should be considered in future studies to validate these findings in a model recreating more precisely the CRC microenvironment. Furthermore, CRC development, progression and tumor-immune response are highly dependent on the genetic profile of the tumor. Thus, future studies using models which recreate different driver-mutation phenotypes will be needed to determine whether the PTPN23-inhibitory effect is tumor-specific or broadly observable in CRC.

Finally, validation in larger patient cohorts and additional experimental models will be required to establish the clinical relevance of PTPN23 as a biomarker and therapeutic target in CRC.

## 4. Materials and Methods

### 4.1. Human Samples

Paraffin-embedded human colon cancer tissue used for PTPN23 staining was obtained from University Hospital Zurich in Switzerland. Ethical approval was obtained from the Cantonal Ethics Committee of the Canton Zürich (PB_2019-00169, first approval date on 1 March 2010) and written informed consent was obtained from all patients before sample collection. The samples were anonymized before analysis.

### 4.2. Mice

Wildtype (WT) BALB/cJRj and C57BL/6JRj mice aged 8–12 weeks were purchased from Janvier Labs (Saint-Berthevin, France). Animals were allowed a 2-week acclimation period under specific-pathogen-free conditions and provided with food and water *ad libitum*. A maximum of 5 animals were housed in individually ventilated cages (IVCs) type 2 long, provided with environmental enrichment, including nesting material and shelter, automated watering system (AWS) and safe aspen premium hygiene bedding. The experimental unit was a single animal. Animal experiments were performed according to animal license ZH083/19 approved by the Animal Welfare Office of the Veterinary Office of the Canton of Zurich, Switzerland. Sample size was calculated according to the approved animal protocol. Our study used confirmatory experiments, so the null hypothesis was that there was no difference between the mean of all groups. The alternative hypothesis was that at least one of the paired comparisons would show a significant difference. In an experiment with *n* groups, we performed stratified randomization based on weight one day before day 0. The mice were weighed and then sorted and grouped into *n* groups. The heaviest *n* mice were randomly assigned to the *n* groups. The remaining mice were randomly assigned to the *n* groups. This procedure was repeated until all mice were divided into the *n* groups.

### 4.3. Subcutaneous Cell Injection

Only females were included in the study based on the practical conditions on the subcutaneous tumor model. Males generally exhibit a thicker subcutaneous layer which increases the difficulty of the injection and, additionally, display fighting behaviors, likely resulting in skin wounds. WT mice were injected with transduced MC38 or CT26 cells diluted 1:2 in matrigel. Tumor growth was monitored every other day and tumor volume was calculated by applying the formula: 4/3 × 3.14 × length/2 × (width/2)^2^, where the shorter dimension was used as width and depth. The mice were euthanized 14 or 21 days after tumor cell injections or earlier if the humane endpoint was reached (>1.5 cm^3^). In any case the maximum permitted tumor burden was not exceeded. On the final day of the experiment, tumor weight was measured as a primary outcome. Monitoring of the animals throughout the experiment, sample collection and analysis at the end of the experiment were conducted in a blind manner.

### 4.4. Cell Culture

*Overexpression*. CT26 cell line was obtained from Prof. Lubor Borsig (Institute of Physiology; University of Zurich, Switzerland) and MC38 cell line was obtained from Prof. Anne Müller (Institute of Molecular Cancer Research; University of Zurich, Switzerland). Transfection and transduction were performed as described previously [26]. Briefly, lentiviral plasmids were transfected into HEK293 cells using Lipofectamine 3000 (Thermo Fisher Scientific, Waltham, MA, USA) and the resulting lentiviral-containing medium was subsequently collected and used to transduce the target cells. Transduction efficiency was evaluated after 72 h based on the expression of the introduced construct.

Transfected CT26 and MC38 cells were grown in Dulbecco’s Modified Eagle’s medium (DMEM) supplemented with 10% FCS, 1% sodium pyruvate and 1% non-essential amino acids with Blasticidin and maintained at 37 °C in a humidified incubator containing 5% CO_2_ (Supplementary Table S1). All experiments were performed with mycoplasma-free cells. PTPN23 expression was verified prior to each experiment to ensure stable overexpression.

### 4.5. Proliferation Assay

Transfected cells were seeded at a concentration of 2 × 10^3^ cells/well in 100 μL culture medium. MTT assay was performed after 24 h (reference group), 48 h and 72 h following the manufacturer’s instructions (Supplementary Table S1). Erlotinib hydrochloride was used at different concentrations to inhibit EGFR. Cells were stimulated by recombinant EGF protein (Supplementary Table S1).

### 4.6. RNA and Protein Measurement

Cells were manually homogenized using a pipette. After RNA isolation and RT-PCR, quantitative real-time PCR was conducted using TaqMan Probes (Supplementary Table S1) on a Quant Studio 6 Flex Thermocycler (Thermo Fisher Scientific, Waltham, MA, USA). Relative expression levels were analyzed with the ΔΔCt method.

Protein extraction and wet Western blotting were carried out according to standard protocols [27]. Details and concentrations of the antibodies used are listed in Supplementary Tables S1 and S2. WesternBright ECL (Advansta, San Jose, CA, USA) was applied to the membranes, and immunoreactive proteins were visualized using a Fusion Solo S Imager (AnimaLab, Poznań, Poland). Gel analysis was conducted with ImageJ software (version 1.54p, National Institutes of Health). Protein quantification was assessed by using ImageJ software.

### 4.7. Histology

The tissue was fixed and embedded in paraffin blocks. Haematoxylin and eosin (H&E) staining was conducted following standard protocols. For immunohistochemistry (IHC), tissues were deparaffinized, and antigen retrieval was performed with antigen unmasking solution at pH 6.0 (Supplementary Table S1) at 98 °C for 30 min. The demasking step was achieved for Cleaved Caspase 3 by using a pressure cooker at 125 °C for 2.5 min. To inhibit endogenous peroxidases, slides were incubated in 0.9% hydrogen peroxide for 15 min at room temperature. Non-specific binding was blocked with 2.5% horse serum for Cleaved Caspase 3, EGFR and CD45 staining, and with a 3% BSA blocking solution for PTPN23 (overnight), Ki67 (1 h) and CD8 (1 h) staining. Primary antibodies (Supplementary Table S2) were diluted in blocking solutions, and slides were incubated overnight at 4 °C. ImmPRESS HRP-labeled antibody (Supplementary Table S1) was applied to IHC samples for 1 h at room temperature, with visualization achieved using the ImmPACT DAB Kit (Vector Laboratories, Newark, CA, USA) (Supplementary Table S1). Tissue sections were analyzed with the Zeiss Axio Imager Z2 and Zeiss 2.6 (Blue Edition) software. Quantification was performed by calculating the proportion of positive cells relative to the total number of cells. ImageJ software (version 1.54p, National Institutes of Health) was used to this end.

### 4.8. Flow Cytometry

Tumor cells were isolated as indicated [28]. First, tumor cells were incubated in PBS containing a viability marker for 30 min at 4 °C in the dark. This was followed by incubation with primary antibodies (Supplementary Table S2) for 20 min at 4 °C in the dark, using FACS buffer (2% FCS, 0.01% NaN_3_, 20% in PBS). Samples were resuspended in PBS and passed through a 70 μm filter. Flow cytometry data were acquired using a BD FACSymphony (Becton, Dickinson and Company, Franklin Lakes, NJ, USA).

### 4.9. Human Datasets

CRC datasets were obtained from publicly available resources [13,29,30,31]. Protein–protein interaction network was generated using STRING by integrating PTPN23 with CRC-associated genes derived from the KEGG pathway dataset [29]. PTPN23 dependency scores were obtained from the DepMap Chronos dataset and analyzed in intestinal cancer cell lines [30]. Single-cell RNA-sequencing data were obtained from a published human CRC atlas, while survival and gene-correlation analyses were performed using GEPIA2 based on TCGA and GTEx data [13,31]. Regarding the CRC patients included in the study, all of the patients included were diagnosed with CRC and signed the informed consent prior to sample collection.

### 4.10. Statistical Analysis

All statistical analyses and visualizations were conducted using GraphPad Prism v.9 (GraphPad Software). When data met the assumptions of normality and equal variance, parametric tests were applied. If these assumptions were not met, non-parametric Mann–Whitney U test was used. Significance levels were set as follows: * *p* < 0.05, ** *p* < 0.01, *** *p* < 0.001, and **** *p* < 0.0001.

## Data Availability

The data presented in this study are available on request from the corresponding author (The data are not publicly available due to privacy and ethical restrictions related to the protection of participant confidentiality).

## Supplementary Materials

The following supporting information can be downloaded at: https://www.mdpi.com/article/10.3390/ijms27177955/s1.

## Abbreviations

The following abbreviations are used in this manuscript:

AWS	automatic watering system
CRC	Colorectal carcinoma
DMEM	Dulbecco’s Modified Eagle’s medium
EGF	Epidermal growth factor
EGFR	Epidermal growth factor receptor
ESCRT	Endosomal sorting complexes required for transport
H&E	Haematoxylin and Eosin
IHC	Immunohistochemistry
IVC	Individually ventilated cage
OE	Overexpression
PTP	Protein tyrosine phosphatase
PTPN23	PTP-Non receptor type 23
PTPs	protein tyrosine phosphatases
WT	wild type
