# Supplementary material for: PTPN23 Overexpression Is Associated with Reduced Proliferation and Tumor Growth in Murine Models of Colorectal Cancer
Source: Int J Mol Sci. 2026 Sep 7;27(17):7955. doi: 10.3390/ijms27177955 (PMC13566123; doi:10.3390/ijms27177955)
Supplement: Supplementary file 1 [file ijms-27-07955-s001.zip › ijms-4499167-supplementary.pdf]

SUPPLEMENTARY MATERIAL

Supplementary Table S1. Chemicals reagents, probes, and recombinant proteins

	Source	Identifier	Concentration
Blasticidine S Hydrochloride	Sigma Aldrich / Merck	15205-25MG	15µg/ml (CT26)<br>5µg/ml (MC38)
BSA	Pan Biotech	PANP06-1391500	5%
Collagenase type IV	Sigma Aldrich	C5138	0.4 mg/mL
DAKO Target Retrieval Solution pH 6.0	Agilent/Dako	S169984-2	
Dispase	Gibco	17105-041	0.6 mg/ml
DMEM Dulbecco's Modified Eagle Medium (D-MEM) (1X), (high glucose)	Thermo Fisher Scientific	31053-028	
Erlotinib hydrochloride	Merck	183319-69-9	10-40 µM
HBSS Hanks Buffer Salt Solution	Sigma Aldrich / Merck	H2387-10	
Histoclear	Brunschwig	HS-200	
Horse Serum	Sigma Aldrich	H0146	2.5%
ImmPACT® DAB Substrate Kit, Peroxidase (HRP)	Vector laboratories	SK-4105	
ImmPRESS® HRP Goat Anti-Rabbit IgG Polymer Detection Kit, Peroxidase	Vector laboratories	MP-7451	
Lipofectamine™ 3000 Transfection Reagent	Thermo Fisher Scientific	L3000015	
Matrigel® Growth Factor Reduced (GFR) Basement Membrane Matrix	Corning	FAL354230	
MEM Non-Essential Amino Acids Solution (100X)	Sigma-Aldrich	M7145	1%
Mouse EGF Recombinant Protein - PeproTech®	Thermo Fisher Scientific	Thermo Fisher Scientific	1 ng/ml
Mouse GAPD (GAPDH) endogenous control (VIC/MGB probe, primer limited)	Thermo Fisher Scientific	A9518-5G	
Mouse PTPN23 real time PCR GEA	Thermo Fisher Scientific	4351370 /<br>Mm01250157_m1	
NaPy 100 mM Sodium Pyruvate	Thermo Fisher Scientific	11360-039	1%
NuPAGE™ LDS-Sample Buffer	Thermo Fisher Scientific	NP0007	
Opti-Mem I Reduced Serum Medium	Thermo Fisher Scientific	31985070	
Pertex	Biosystems Switzerland AG	41-4012-00	
Polybrene, Hexadimethrine bromide	Sigma	H9268-5G	
TaqMan Fast Universal PCR Master Mix (2x)	Thermo Fisher Scientific	4367846	
Thiazolylblau (3-(4,5-Dimehtylthiazolyl-2) -2,5-diphenyl-2H-tetrazoliumbromid) - MTT	Roth	4022.1	
Western Bright ECL horseradish peroxidase substrate	Advansta	K-12045-D50	
Zombie NIR Fixable Viability Kit	BioLegend	423106	1:500

Supplementary Table S2. Antibodies

	Source	Identifier	Application	Dilution
Hamster anti-CTLA4 (CD152) - PE/Cy7	BioLegend	106313	FACS	1:200
Rabbit anti-human PTPN23	Atlas Antibodies	HPA016845	IHC	1:200
Mouse anti-CD8 – PE-CF594	BD Becton Dickinson Pharminge Biosciences	562283	FACS	1:200
Mouse anti-NK1.1 (PK136) - BV711	BioLegend	108745	FACS	1:100
Rabbit anti mouse PTPN23 antibody Polyclonal Antibody	Biozol Diagnostica / MyBioSource	MBS-MBS9603397	WB	1:500
Rabbit anti-CD45 (D378Q)	Cell Signaling	70257-S	IHC	1:100
Rabbit anti-human AKT	Cell Signaling	9272	WB	1:1000
Rabbit anti-human pAKT	Cell Signaling	4058I	WB	1:1000
Rabbit anti-human ERK	Cell Signaling	9102	WB	1:1000
Rabbit anti-human pERK	Cell Signaling	9101S	WB	1:1000
Rabbit anti-cleaved Caspase 3	Invitrogen/Thermo Fisher	PA5-114687	IHC	1:600
Rabbit anti-EGFR (D1P9C)	Cell Signaling	71655	IHC	1:200
Rabbit anti-Ki67	Thermo Fisher Scientific	RM-9106-S0	IHC	1:100
Rat anti-CD279 (PD1) (29F.1A12) - APC	BioLegend	135210	FACS	1:100
Rat anti-CD3 (17A2) – BV785	Lucerna-Chem / BioLegend	100232	FACS	1:200
Rat anti-CD4 (RM4-5) - BV650	BioLegend	100546	FACS	1:200
Rat anti-CD44 (IM7) - FITC	Thermo Fisher Scientific	11-0441-85	FACS	1:200
Rat anti-CD45 (30-F11) - BV510	BioLegend	103138	FACS	1:400
Rat anti-CD45R (B220) - PE-Cy5	Thermo Fisher Scientific	15-0452-83	FACS	1:200
Rat anti-CD62L - BV605	BD Becton Dickinson Pharminge Biosciences	563252	FACS	1:200
Rat anti-CD8a (53-6.7) - PerCP-Cy5.5	Thermo Fisher Scientific / Life Technologies	45-0081-82	FACS	1:200
Rat anti-Tim3 (CD366) - PE	Lucerna-Chem / BioLegend	134004	FACS	1:200

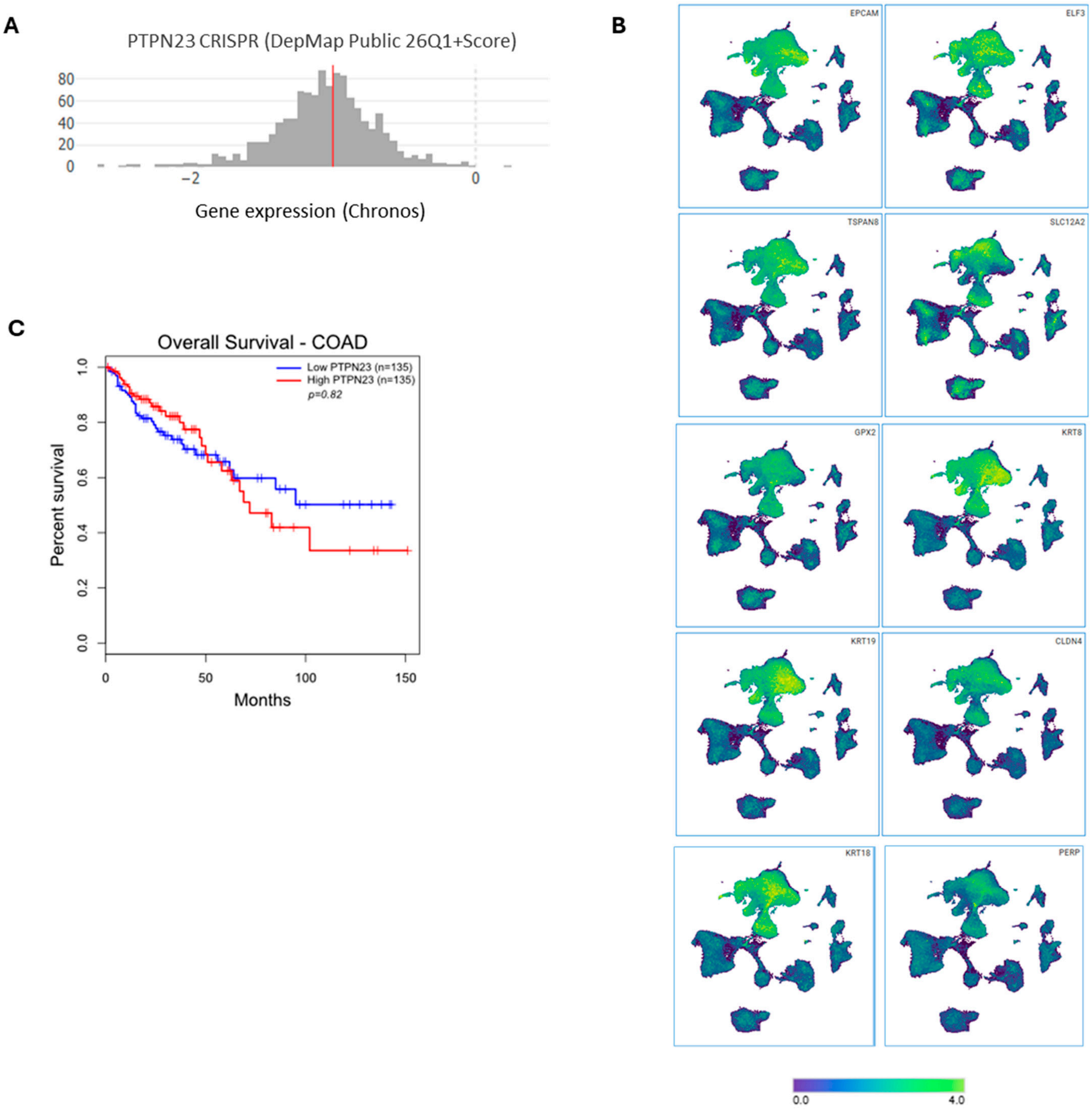

Supplementary Figure S1. PTPN23 expression analysis in colon cancer datasets. *A. DepMap gene effect scores of PTPN23 in cancer cell lines. The red line represents the mean dependency score of pan-essential genes.* ***B.*** *UMAPs of the genes used for annotating malignant cells in the human scRNA sequencing atlas. UMAP cell annotation for each cluster can be found in the original article [14].* ***C.*** *Survival curve from colon adenocarcinoma (COAD) patients stratified into high (red) or low (blue) PTPN23 expression levels (TCGA database).*

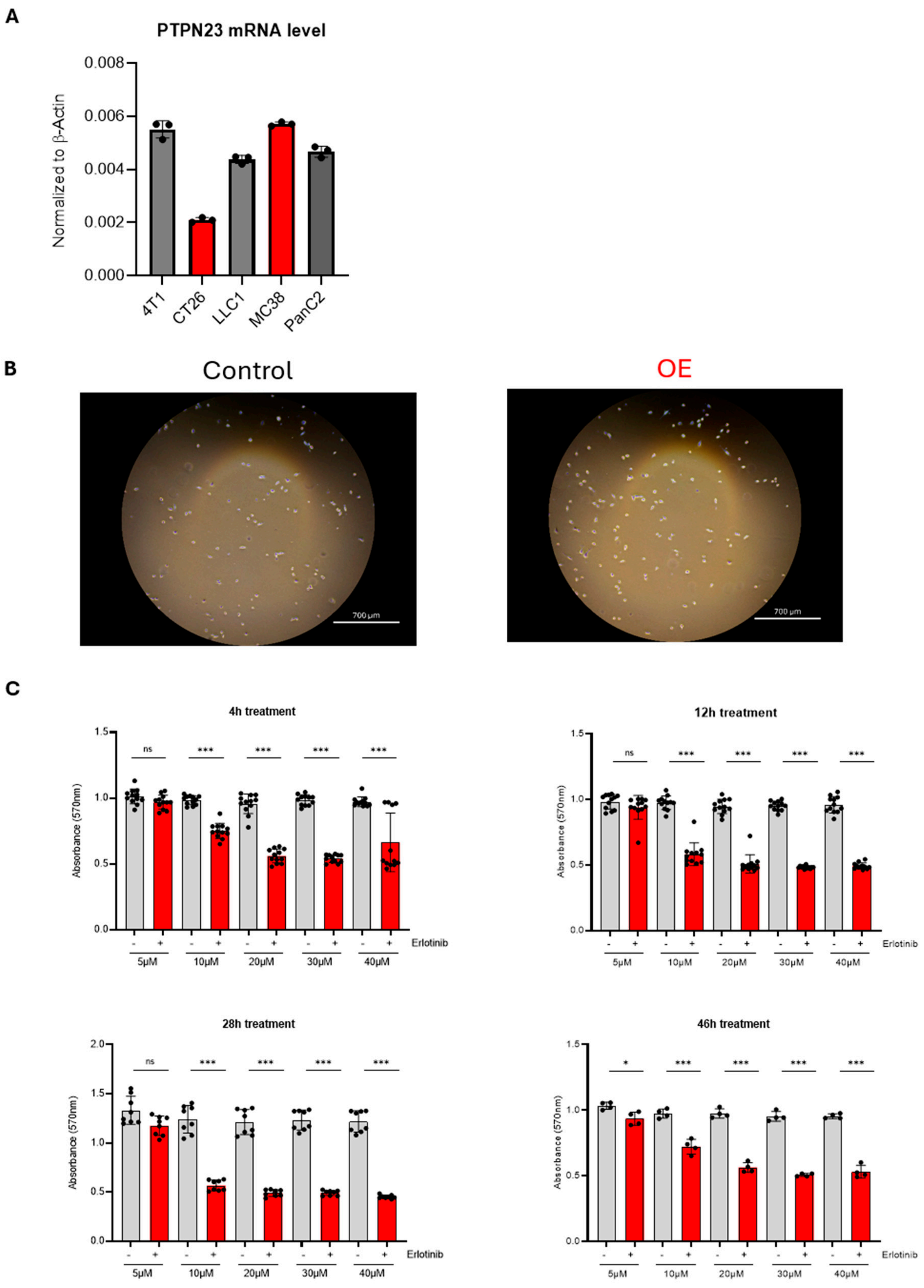

Supplementary Figure S2. PTPN23 expression in multiple cell lines and effects of EGFR inhibition. A. *PTPN23 mRNA expression level measured in 4T1 (breast cancer), LLC1.1 (lung cancer), PanC2 (pancreatic cancer), MC38 and CT26 (colon cancer) at steady state.* ***B.*** *Original images of transduced MC38 cells (50x magnification; scale bar=700 µm).* ***C.*** *Bar plots indicating the absorbance values of MC38 cells treated with Erlotinib (+) or DMSO (-) at different concentrations during 4h, 12h, 28h and 46h treatment. Error bars represent mean ± SD. P-values were determined by one-way ANOVA Test (*= P<0.05, ***P<0.001, ns = non-significant).*

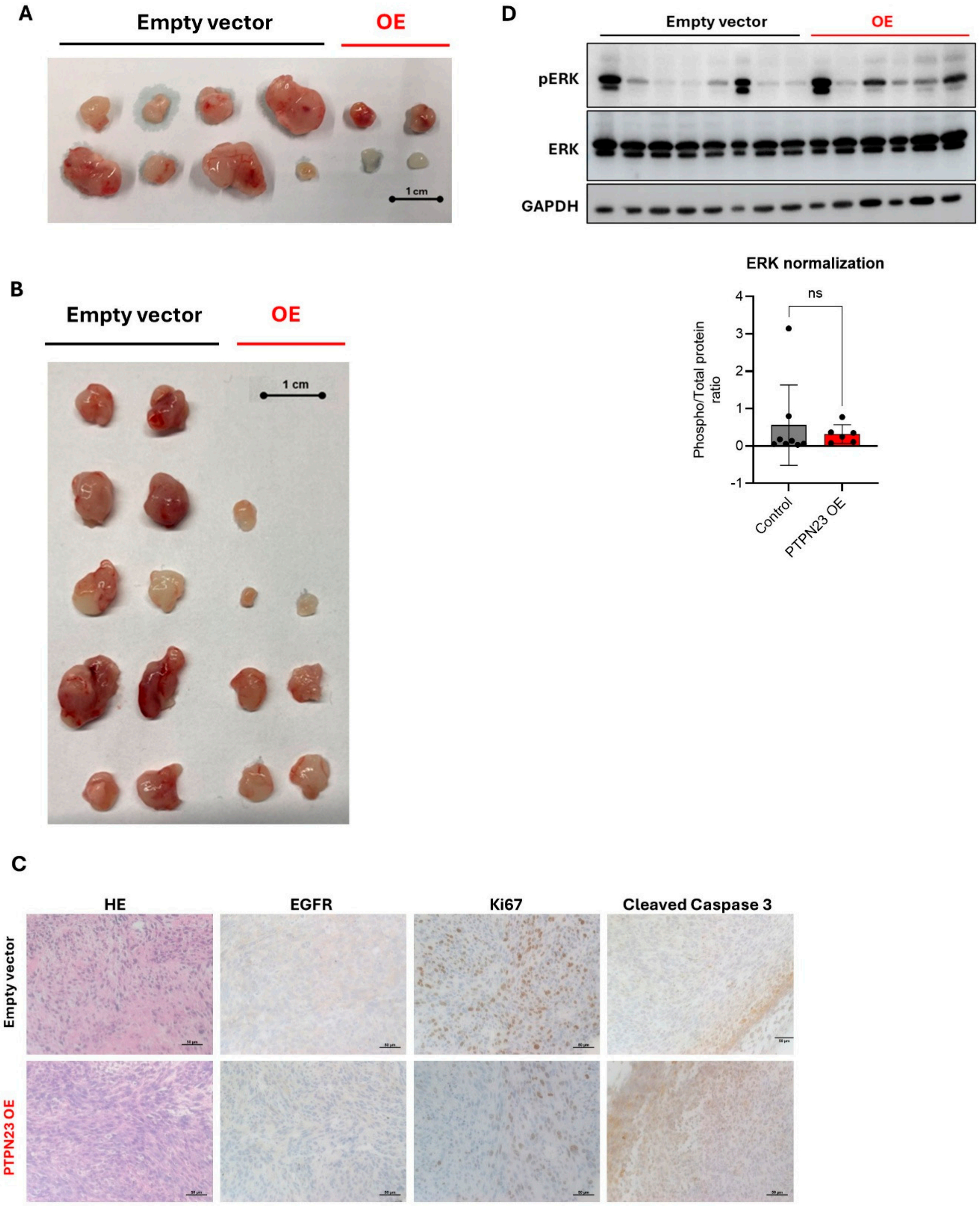

Supplementary Figure S3. Representative tumor images from the subcutaneous tumor model and analysis of ERK signaling. *A. Tumor picture from the control or PTPN23-overexpressing (OE) MC38-subcutaneous cell injections in C57BL/6JRj mice. B. Tumor picture from the control or PTPN23-overexpressing (OE) CT26-subcutaneous cell injections in BALB/cJRj mice. C. HE and IHC staining for cleaved Caspase-3, Ki67, and EGFR in CT26 tumors from control or PTPN23-overexpressing groups (200× magnification). D. Western blot and protein quantification of total protein ERK (44-46 kDa) and phosphorylated-ERK (44-46 kDa) from CT26-tumor model from empty-vector group (n=8) and PTPN23-overexpressing group (OE; n=6). GAPDH (37 kDa) was used as a loading control. One experiment was performed.*

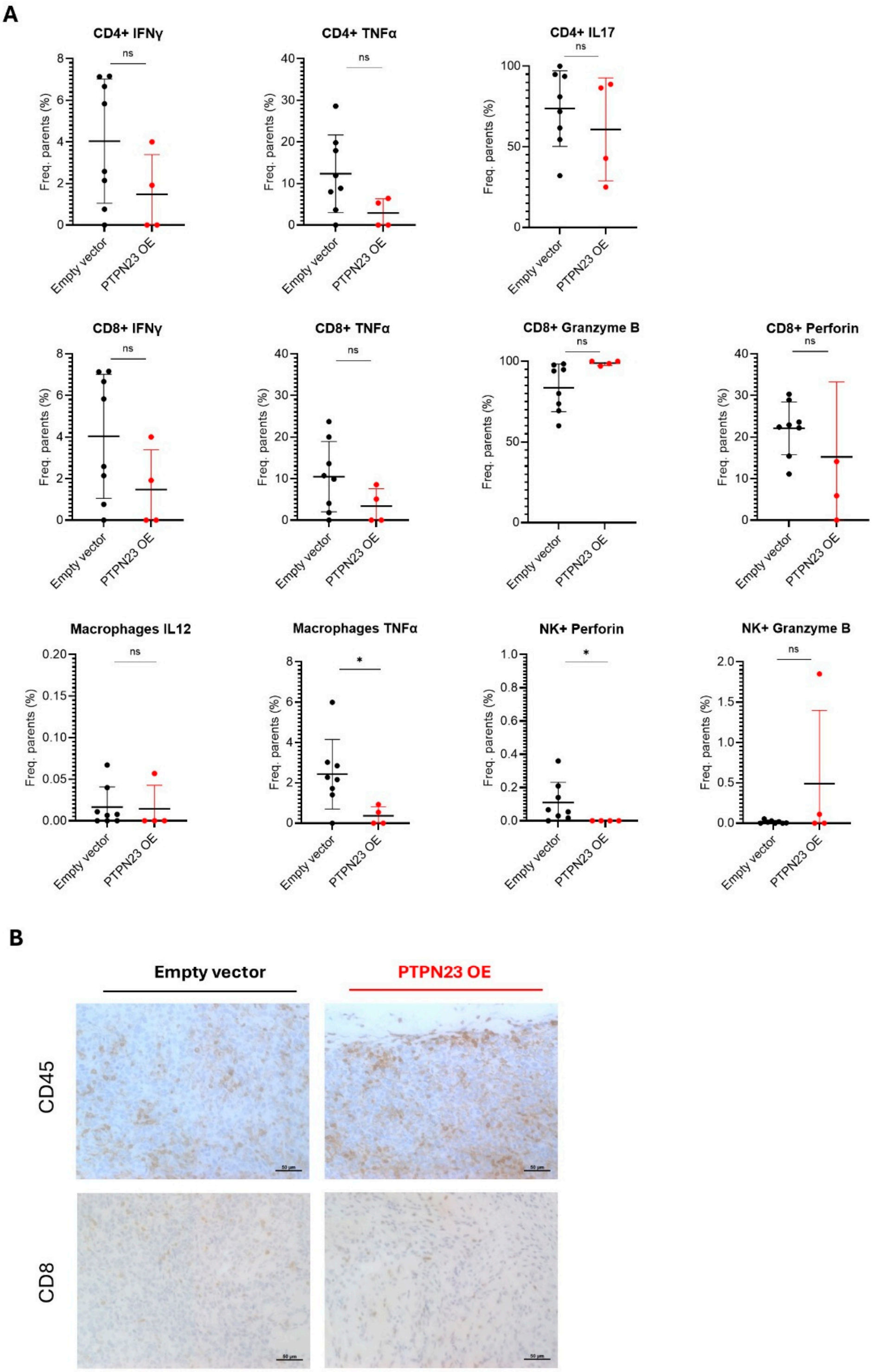

Supplementary Figure S4. Flow cytometry and immunohistochemistry analyses show no significant differences between groups. A. *Frequencies of parents of different immune populations from tumor tissue of empty-vector or PTPN23-overexpression (OE) cell injections.* ***B.*** *IHC staining for CD45+ and CD8+ in CT26 tumors from control or PTPN23-overexpressing groups (200× magnification). Data from one experiment consisting of control group (n=5) and PTPN23 overexpressing group (n=5). Error bars represent mean ± SD. P-values were determined by 2-tailed Mann–Whitney U Test (*= P<0.05, ***P<0.001, ns = non-significant).*
